# Effects of Aging on Intramuscular Collagen-Related Factors After Injury to Mouse Tibialis Anterior Muscle

**DOI:** 10.3390/ijms26020801

**Published:** 2025-01-18

**Authors:** Yuji Kanazawa, Tatsuo Takahashi, Takao Inoue, Mamoru Nagano, Satoshi Koinuma, Haruki Eiyo, Yuma Tamura, Ryo Miyachi, Naoya Iida, Kenichiro Miyahara, Yasufumi Shigeyoshi

**Affiliations:** 1 Department of Physical Therapy, Hokuriku University, Kanazawa 920-1180, Japan; ry_miyachi@hokuriku-u.ac.jp (R.M.); k-miyahara@hokuriku-u.ac.jp (K.M.); 2 Well-Being Research Team, Hokuriku University, Kanazawa 920-1180, Japan; t-takahashi@hokuriku-u.ac.jp; 3 Department of Anatomy and Neurobiology, Faculty of Medicine, Kindai University, Osakasayama 589-8511, Japan; m-nagano@med.kindai.ac.jp (M.N.); koi@med.kindai.ac.jp (S.K.); iida.naoya@med.kindai.ac.jp (N.I.); shigey@med.kindai.ac.jp (Y.S.); 4 Department of Clinical Pharmacology, Hokuriku University, Kanazawa 920-1181, Japan; 5 Department of Pathology, Faculty of Medicine, Kindai University, Osakasayama 589-8511, Japan; takao@med.kindai.ac.jp; 6 Department of Rehabilitation, Dokkyo Medical University Nikko Medical Center, Nikko 321-1298, Japan; h-eiyou@dokkyomed.ac.jp (H.E.); ytamura@dokkyomed.ac.jp (Y.T.)

**Keywords:** aging, muscle injury, extracellular matrix, lysyl oxidase, matrix metalloproteinase

## Abstract

Collagen I is the most abundant type of intramuscular collagen. Lysyl oxidase promotes collagen cross-link formation, which helps stabilize the extracellular matrix. Furthermore, matrix metalloproteinases, responsible for collagen degradation, maintain typical muscle structure and function through remodeling. Although it is well known that aging leads to delayed recovery of muscle fibers, the impact of aging on the remodeling of intramuscular collagen is not well understood. In this study, we investigated the impact of aging on collagen remodeling during muscle injury recovery using young and old mouse models. Muscle injury was induced in the right tibialis anterior (TA) muscle of male C57BL/6J mice [aged 21 weeks (young) and 92 weeks (old)] using intramuscular cardiotoxin injection, with the left TA serving as a sham with saline injection. Following a one-week recovery period, aging was found to delay the recovery of the fiber cross-sectional area. The intensity and area of immunoreactivity for collagen I were significantly increased in old mice compared to young mice post-injury. Additionally, *Lox* expression and the number of LOX (+) cells in the extracellular matrix significantly increased in old mice compared to young mice post-injury. Furthermore, *Mmp9* and MMP9 expression levels after muscle injury were higher in old mice than in young mice. These results suggest that muscle injury in old mice can lead to increased collagen I accumulation, enhanced collagen cross-link formation, and elevated MMP9 expression compared to young mice.

## 1. Introduction

Skeletal muscle fibers are enveloped by an extracellular matrix (ECM), a complex meshwork composed of collagen, glycoproteins, proteoglycans, and elastin [1,2,3]. The ECM in skeletal muscle consists of several layers: the outermost epimysium, followed by the perimysium, endomysium, and basement membrane [3,4]. This matrix plays crucial roles in muscle protection, regeneration, and transmission of contractile forces of muscle fibers [5,6,7,8,9,10,11]. Collagen is a predominant component of the ECM, with type I being most abundant in the epimysium, perimysium, and endomysium [11,12]. Collagen I provides tensile strength and stiffness to muscle fibers [13]. In intramuscular collagen, collagen I is a major component and plays an important role in normal muscle structure and function.

Previous studies on ECM remodeling during muscle injury recovery have highlighted collagen expression as a crucial step [8,14,15]. Matrix metalloproteinases (MMPs), enzymes that degrade collagen, also play significant roles in skeletal muscle ECM remodeling [16]. The expression of *Mmp 2*, *9*, and *14* is induced during muscle recovery processes [15,16], and an ongoing imbalance between collagen production and degradation is implicated in pathological conditions involving excessive collagen accumulation during wound healing [16]. Therefore, collagen and MMP induction and expression in the ECM are critical for muscle recovery post-injury.

The lysyl oxidase (LOX) family comprises five members, including LOX, lysyl oxidase-like 1 (LOXL1), LOXL2, LOXL3, and LOXL4, among which LOX contributes to ECM functionalization by promoting the formation of cross-links between ECM components [17]. LOX, which is involved in collagen cross-link formation, is also known to be strongly associated with fibrosis and post-injury repair in various organs [18]. Furthermore, LOX is reportedly involved in both the ECM and muscle differentiation [19]. Thus, it is a key factor in intramuscular collagen remodeling and muscle recovery post-injury. In fact, LOX expression is known to be highly expressed in the skeletal muscles of mdx mice, which exhibit fragile muscle structures [20]. However, the effects of aging on LOX expression and localization during the collagen remodeling process remain unresolved. To this end, in this study, we examined the effects of aging on collagen remodeling and the expression and localization of LOX during recovery from muscle injury using old and young mouse models.

## 2. Results

### 2.1. Muscle Function

**Figure 1:**
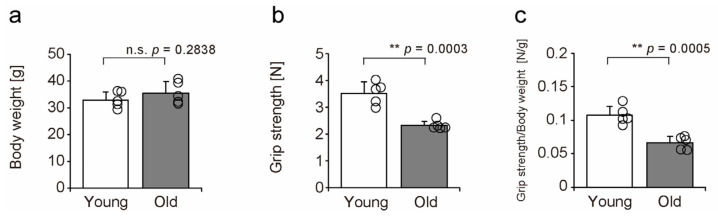
Differences in body weight (**a**), grip strength (**b**), and grip strength normalized by body weight (**c**) between young and old mice before the experiment. Data are expressed as mean ± standard deviation. Statistical significance was determined using Student’s *t*-test (**a**–**c**). Individual data points are shown as circles on the graph for each group (n = 5 per group). Differences between groups were considered significant when *p* < 0.05. ** *p* < 0.001, n.s. indicates no statistically significant difference. One week before the induction of muscle injury, grip strength and body weight were measured using five 20-week-old young mice and five 90-week-old old mice.

Prior to the experiment, a grip strength test was performed to determine the differences in muscle function between young and old mice (Figure 1). No difference in body weight was noted between young and old mice (Figure 1a). Aging decreased the grip strength and grip strength normalized by body weight (Figure 1b,c). The grip strength of mice has been reported to decrease with age [21]. These results confirmed that muscle function in the old mice used in this study declined with age.

### 2.2. Body and Muscle Weight

**Figure 2:**
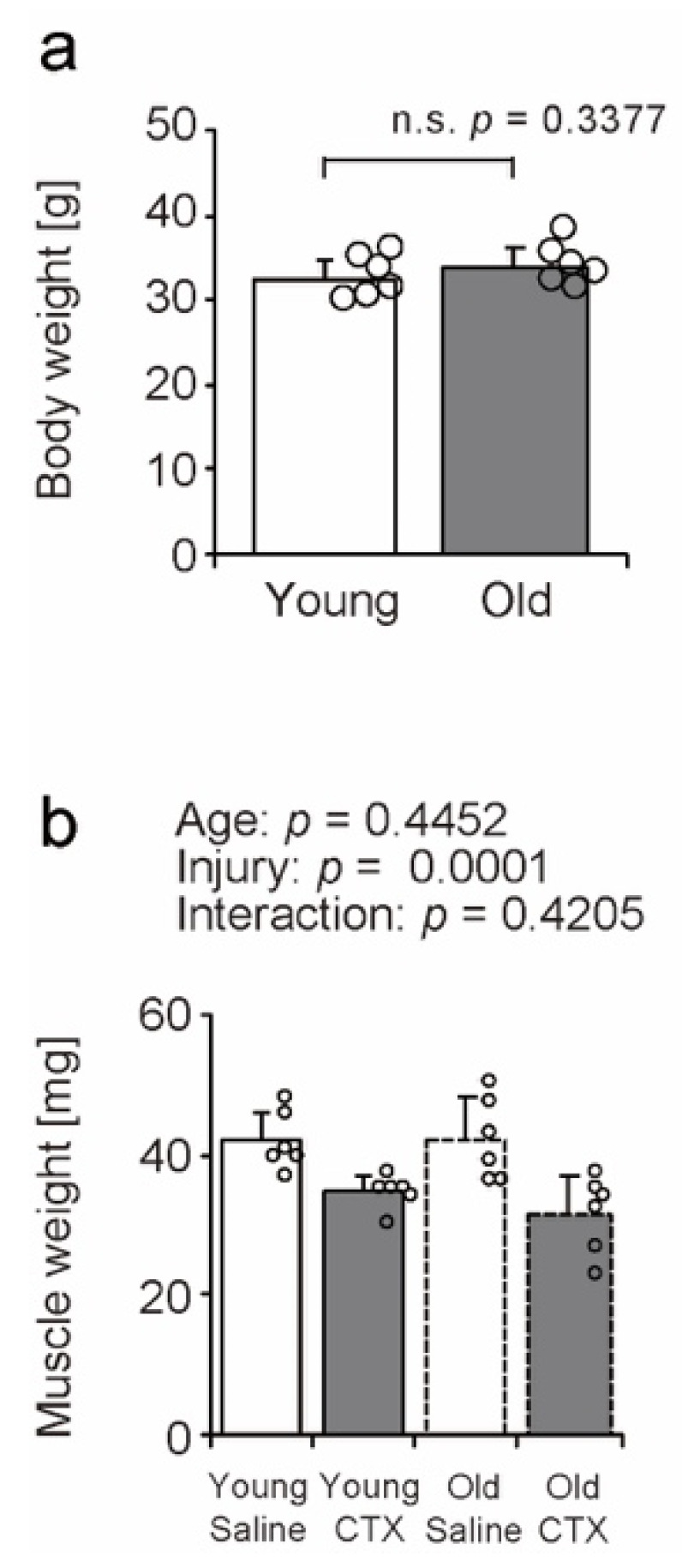
Differences in body weight (**a**) and tibialis anterior muscle weight (**b**) between young and old mice. Data are expressed as mean ± standard deviation, with n = 6 animals per group. To compare the effects of aging between young and old mice, statistical significance was determined using the Student’s *t*-test (**a**). The statistical interaction between age and muscle injury was evaluated using a two-way analysis of variance, which allows for the assessment of both main effects (age and injury) and their statistical interaction (**b**). Individual data points are shown as circles on the graph for each group. Differences between groups were considered significant when *p* < 0.05 unless otherwise indicated. n.s.: Denotes no statistically significant difference. CTX: cardiotoxin. The right tibialis anterior muscle of young mice is designated as Young + CTX, and the left tibialis anterior muscle is designated as Young + Saline. The right tibialis anterior muscle of old mice is designated as Old + CTX, and the left tibialis anterior muscle is designated as Old + Saline.

To investigate the impact of aging and muscle injury on the tibialis anterior (TA), we measured body and muscle weight (Figure 2). There was no significant difference in body weight between young and old mice (Figure 2a). Muscle weight decreased following muscle injury in both young and old age groups (Figure 2b). Muscle weight loss associated with muscle injury has been reported by a previous study [22]. Changes in muscle weight in the muscle injury model were confirmed in this study, allowing for subsequent analysis.

### 2.3. Muscle Injury

**Figure 3:**
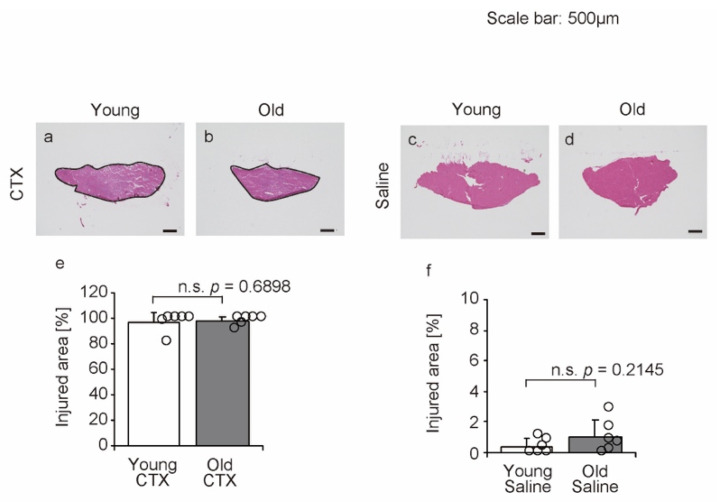
Hematoxylin and eosin staining of tibialis anterior muscles and injured areas. Representative cross-sections of the tibialis anterior muscle are shown (**a**–**d**). The scale bar represents 500 µm. Injured areas where fibers with central nuclei or necrotic fibers were measured in the images (within the black lines in (**a**,**b**)). Data are presented as the mean ± standard deviation (n = 6 per group). Significant differences between young and old mice were determined using Mann–Whitney U test (**e**) and Student’s *t*-test (**f**). n.s.: not significant. All data are represented as circles in the graph. CTX: cardiotoxin. The right tibialis anterior muscle of young mice is designated as Young + CTX, and the left tibialis anterior muscle is designated as Young + Saline. The right tibialis anterior muscle of old mice is designated as Old + CTX and the left tibialis anterior muscle is designated as Old + Saline.

To confirm the muscle injury caused by cardiotoxin (CTX) injection, the injured areas having fibers with central nuclei or necrotic fibers were observed 1 week after muscle injection in hematoxylin and eosin (HE)-stained transverse sections at the muscle belly level of the tibialis anterior muscle (within the black lines in Figure 3a,b). In both young and old mice, the average percentage of the injured area in the entire transverse section was more than 95% and did not differ by age (Figure 3e). Thus, drug-induced muscle injury was extensive in both young and old mice, allowing for further investigation. In addition, the effect of saline injection was confirmed (Figure 3c,d). Both young and old mice had an average injured area of less than 1%, with no age-related differences (Figure 3f). It was confirmed that CTX induces muscle injury in this study.

### 2.4. Fiber Cross-Sectional Area

**Figure 4:**
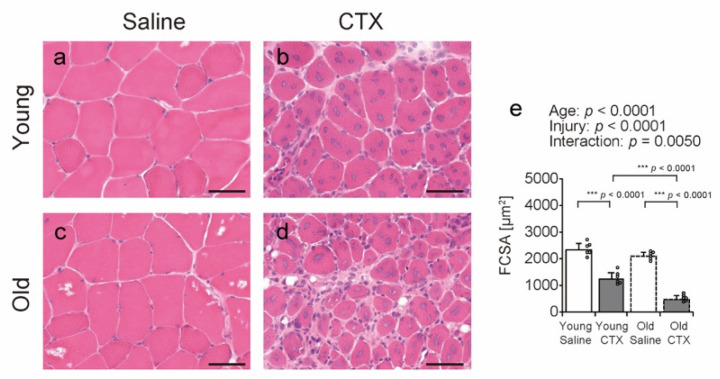
Hematoxylin and eosin (HE) staining of tibialis anterior muscles and fiber cross-sectional area (FCSA). Transverse sections of the tibialis anterior muscle were prepared and stained with HE to assess muscle structure and morphology (**a**–**d**). The scale bar represents 50 µm. The FCSA (**e**) was quantified from the images. The data are expressed as mean ± standard deviation, with n = 6 per group. A two-way analysis of variance was performed to examine the statistical interaction between aging and muscle injury (**e**). Post hoc comparisons between all groups were conducted using Tukey’s honestly significant difference test (**e**). Statistical significance is indicated by *** *p* < 0.0001. Individual data points are represented as circles in the graph. CTX: cardiotoxin. The right tibialis anterior muscle of young mice is designated as Young + CTX, and the left tibialis anterior muscle is designated as Young + Saline. The right tibialis anterior muscle of old mice is designated as Old + CTX, and the left tibialis anterior muscle is designated as Old + Saline.

To assess the effects of aging and muscle injury on TA muscle fibers, we measured the fiber cross-sectional area (FCSA; Figure 4a–e). Due to a significant statistical interaction between age and injury for FCSA (Figure 4e), multiple comparisons were conducted across all groups. FCSAs in the Young + CTX and Old + CTX groups were significantly lower than those in the Young + Saline and Old + Saline groups (Figure 4e). Additionally, FCSA in the Old + CTX group was significantly lower than that in the Young + CTX group (Figure 4e). These findings suggest that aging may delay the recovery of the FCSA following muscle injury. In a previous study, the FCSA in regenerating muscle of old mice was also reported to have delayed recovery compared to young mice [22]. These results of the previous study and the present study suggest that aging may delay the recovery of the FCSA after muscle injury.

### 2.5. Collagen-Related Factors

**Figure 5:**
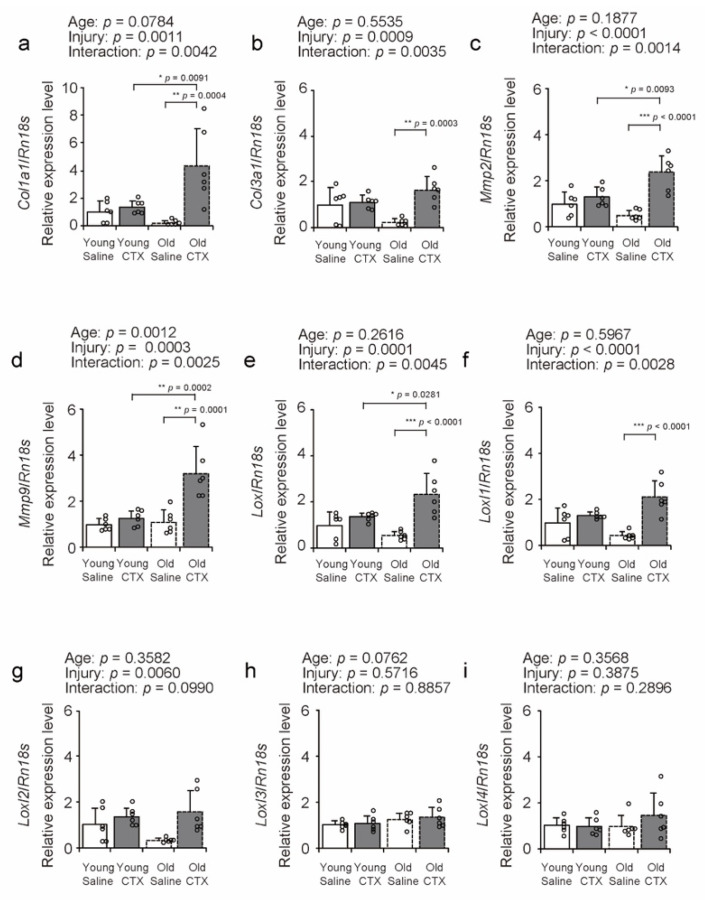
Comparison of collagen-related factors. Relative mRNA expression levels of *Col1a1* (**a**), *Col3a1* (**b**), *Mmp2* (**c**), *Mmp9* (**d**), *Lox* (**e**), *Loxl1* (**f**), *Loxl2* (**g**), *Loxl3* (**h**), and *Loxl4* (**i**) in the tibialis anterior muscles. Expression levels were normalized to 18S ribosomal RNA (*Rn18s*). Data are expressed as mean ± standard deviation, with n = 6 per group. A two-way analysis of variance was performed to assess the statistical interaction between aging and muscle injury across panels (**a**–**i**). When significant statistical interaction were detected, Tukey’s honestly significant difference test was used for post hoc comparisons between all groups (**a**–**f**). Statistical significance is indicated as *** *p* < 0.0001, ** *p* < 0.001, * *p* < 0.05, and n.s. indicates no significant difference. Individual data points are shown as circles in the graph. CTX: cardiotoxin. The right tibialis anterior muscle of young mice is designated as Young + CTX, and the left tibialis anterior muscle is designated as Young + Saline. The right tibialis anterior muscle of old mice is designated as Old + CTX, and the left tibialis anterior muscle is designated as Old + Saline.

To examine the influence of aging and muscle injury on collagen-related factors, we measured the expression of *Col1a1*, *Col3a1*, *Mmp2*, *Mmp9*, *Lox*, *Loxl1*, *Loxl2*, *Loxl3*, and *Loxl4* using quantitative PCR (Figure 5a–i). Muscle injury increased the expression of these genes other than *Loxl3* and *Loxl4*, and significant statistical interactions between age and injury were observed for these genes other than *Loxl2, Loxl3*, and *Loxl4*, prompting multiple comparisons among all groups. *Col1a1*, *Col1a3, Mmp2*, *Mmp9*, *Lox*, and *Loxl1* expression levels were significantly higher in the Old + CTX group compared to those in the Old + Saline group (Figure 5a–f), and the *Col1a1*, *Mmp2*, *Mmp9*, and *Lox* expression levels in the Old + CTX group were also higher than those in the Young + CTX group (Figure 5a,c,d,e). These results suggest two possibilities. The first is that aging may promote the expression of *Col1a1*, *Col1a3, Mmp2*, *Mmp9*, and *Lox* mRNAs after muscle injury. The second is that aging tends to reduce steady-state levels of *Col1a1*, *Col1a3, Mmp2*, *Mmp9*, and *Lox* mRNAs, indicating that the ability to induce genes associated with muscle injury may have been maintained.

### 2.6. Collagen I Localization

**Figure 6:**
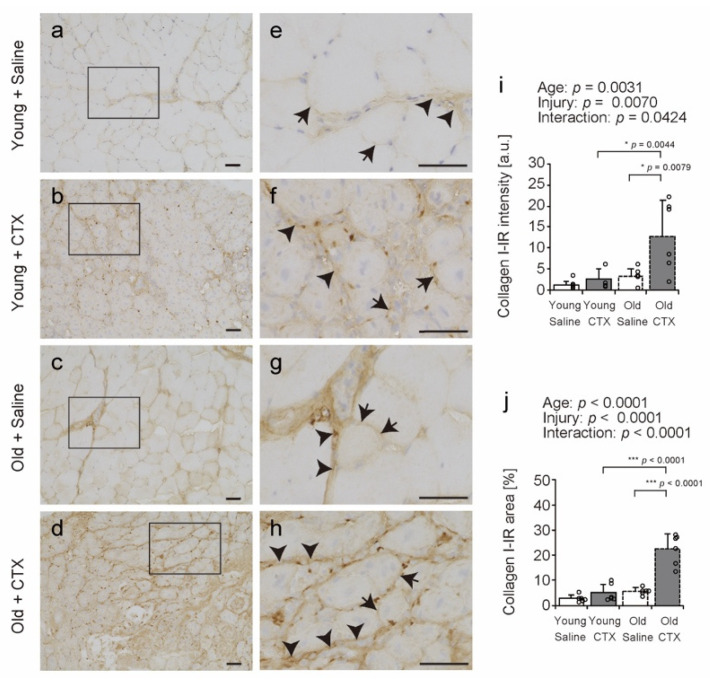
Collagen I localization. Cross-sections of tibialis anterior muscles were stained with anti-collagen I (**a**–**h**) antibody. Hematoxylin was used for counterstaining (**a**–**h**). (**e**–**h**) Magnified views of the rectangular regions outlined in panels (**a**–**d**) provide a closer examination of the selected areas. Arrows and arrowheads indicate representative localization of collagen I (**e**–**h**). Collagen I-immunoreactivity (IR) was predominantly observed in the endomysium (arrow) and perimysium (arrowhead) in all groups (**e**–**h**). Scale bar = 50 μm. The intensity and area of collagen I-IR were quantified (**i**,**j**). Data are expressed as the mean ± standard deviation, with n = 6 per group. A two-way analysis of variance was performed to evaluate the statistical interaction between age and injury (**i**,**j**). A significant statistical interaction between age and injury influenced collagen I-IR intensity and area, prompting multiple group comparisons by Tukey’s honestly significant difference test (**i**,**j**). Statistical significance is indicated by *** *p* < 0.0001, * *p* < 0.05. Individual data points are shown as circles in the graph. CTX: cardiotoxin. The right tibialis anterior muscle of young mice is designated as Young + CTX, and the left tibialis anterior muscle is designated as Young + Saline. The right tibialis anterior muscle of old mice is designated as Old + CTX, and the left tibialis anterior muscle is designated as Old + Saline.

Since collagen I is the most abundant collagen in muscle [11,12], and since *Col1α1* expression was markedly elevated in the injured senile muscle in this study, we observed TA cross-sections using immunohistochemistry (IHC) with an anti-collagen I antibody (Figure 6a–h). Collagen I-immunoreactivity (IR) was predominantly observed in the endomysium and perimysium of the TA in all groups (arrow and arrowhead in Figure 6e–h). The intensity and area of collagen I-IR were measured using stained images. A significant statistical interaction between age and injury influenced collagen I-IR intensity and area (Figure 6i,j), prompting multiple group comparisons. Collagen I-IR intensity and area in the Old + CTX group was higher than that in the Old + Saline and Young + CTX groups. In a previous study, it was reported that the positive areas of collagen I in the muscles of old mice are enlarged after muscle injury compared to those in young mice [22]. The results of both the previous study and the present study suggest that aging may enhance collagen I accumulation following muscle injury in the ECM region.

### 2.7. MMP2 and MMP9 Expression Levels

**Figure 7:**
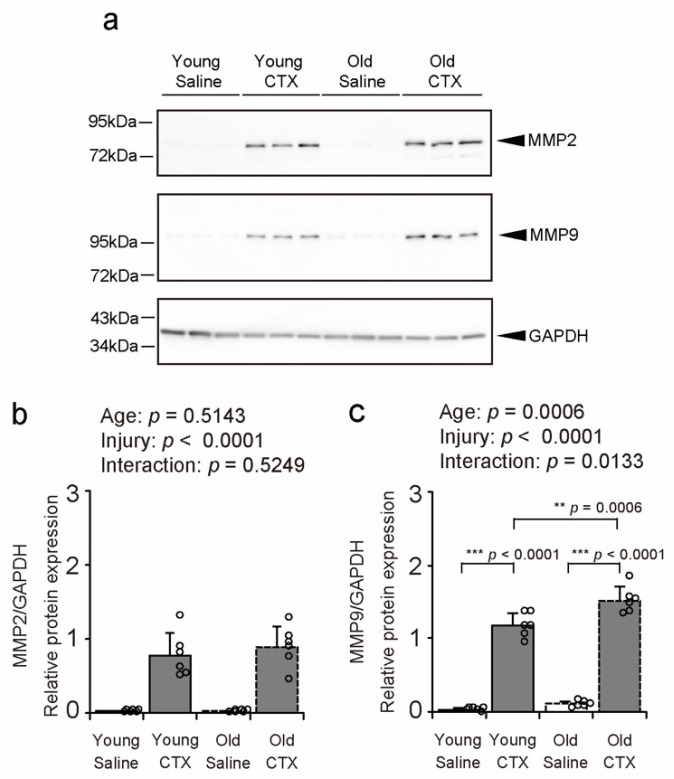
Matrix metalloproteinase (MMP) 2 and MMP9 expression levels. Representative western blot (**a**) and protein expression levels of MMP2 (**b**) and MMP9 (**c**) in the tibialis anterior muscle. Data are expressed as mean ± standard deviation, with n = 6 per group. A two-way analysis of variance was performed to assess the statistical interaction between aging and muscle injury (**b**,**c**). Tukey’s honestly significant difference test was used for post hoc comparisons between all groups (**c**). Statistical significance is indicated by *** *p* < 0.0001, ** *p* < 0.001, and n.s. denotes no significant difference. Individual data points are represented as circles in the graph. CTX: cardiotoxin. The right tibialis anterior muscle of young mice is designated as Young + CTX, and the left tibialis anterior muscle is designated as Young + Saline. The right tibialis anterior muscle of old mice is designated as Old + CTX, and the left tibialis anterior muscle is designated as Old + Saline.

To confirm the expression of MMP2 and MMP9, enzymes involved in collagen degradation, in the TA after muscle injury, their expression levels were assessed using WB (Figure 7a). MMP2 expression increased following muscle injury (Figure 7b). Age significantly interacted with injury in MMP9 expression levels (Figure 7c), prompting multiple comparisons across all groups. MMP9 expression in the Young + CTX group was significantly higher than that in the Young + Saline group (Figure 7c). Additionally, MMP9 expression in the Old + CTX group was significantly higher than that in both the Old + Saline and Young + CTX groups (Figure 7c). These findings indicate that MMP9 expression post-muscle injury can be exacerbated by aging.

### 2.8. LOX Expression

**Figure 8:**
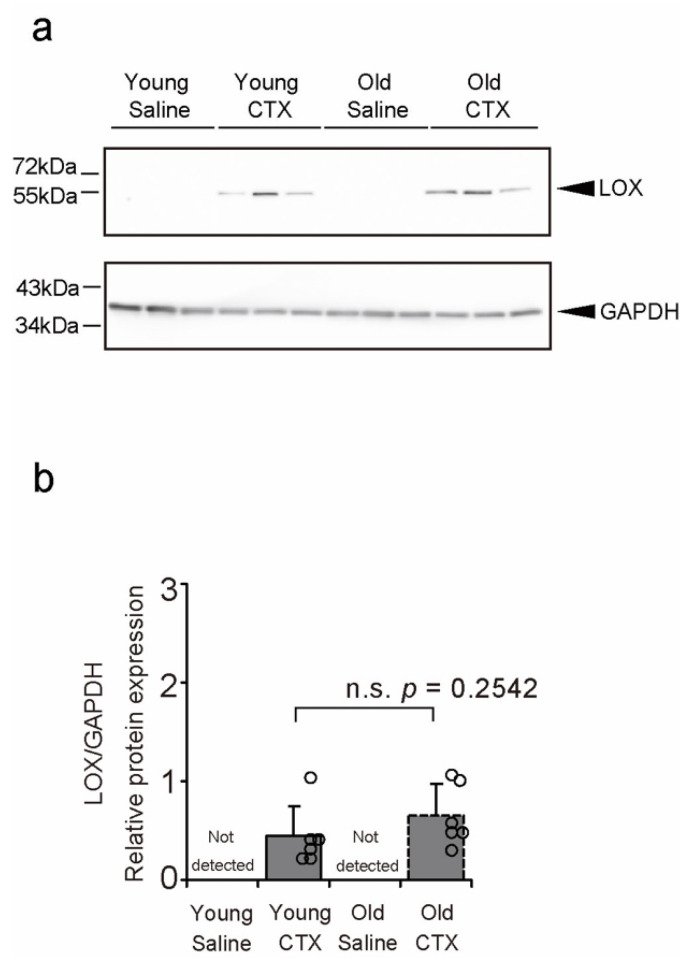
Levels of lysyl oxidase (LOX). The levels were determined using western blotting with an anti-LOX antibody. Representative western blot (**a**) and levels of LOX in the tibialis anterior muscle (**b**). Data are presented as the mean ± standard deviation, n = 6 per group. It was not detected in saline-injected muscle. The detected LOX expression data in the post-injury muscle of young and old mice were compared using the Student’s *t*-test. (**b**). n.s.: not significant. Data are represented as circles in the graph. CTX: cardiotoxin. The right tibialis anterior muscle of young mice is designated as Young + CTX, and the left tibialis anterior muscle is designated as Young + Saline. The right tibialis anterior muscle of old mice is designated as Old + CTX, and the left tibialis anterior muscle is designated as Old + Saline.

The expression of LOX was measured using western blotting (WB; Figure 8a). It was not detected in saline-injected muscle in both young and old mice but was detected in CTX-injected injured muscle in both young and old mice. No age differences were observed in the detected LOX expression levels (Figure 8b). These findings suggest that LOX expression can increase after injury in both young and old mice.

### 2.9. LOX Localization

**Figure 9:**
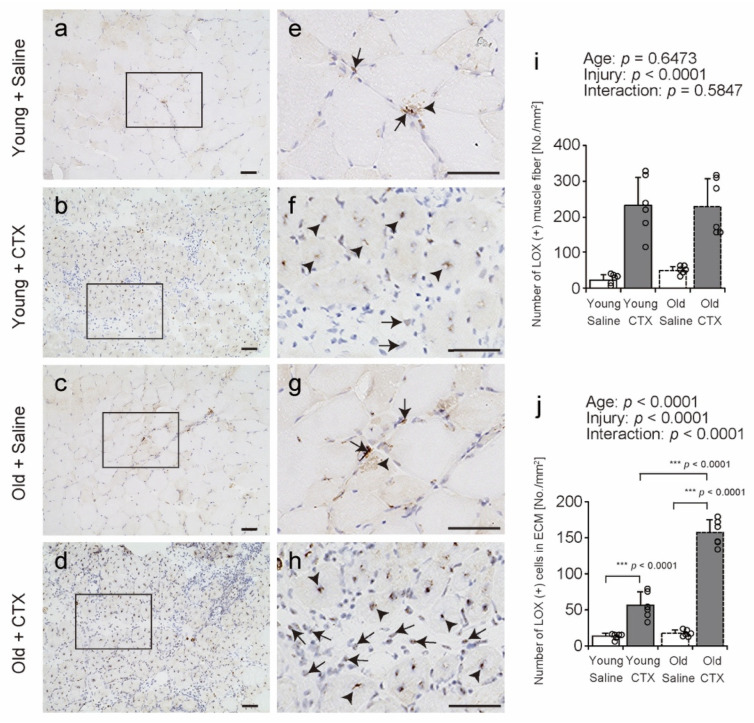
Localization of lysyl oxidase (LOX). Cross-sections of the tibialis anterior muscle were stained with an anti-LOX antibody (**a**–**h**). Hematoxylin was used for counterstaining (**a**–**h**). (**e**–**h**) Enlarged views of the rectangular region outlined in panels (**a**–**d**). Arrows and arrowheads indicate representative localization of LOX (+) cells in the ECM and LOX (+) muscle fibers (**a**–**h**). Scale bar = 50 μm. The number of LOX (+) muscle fibers and LOX (+) cells in the ECM per area were counted (**i**,**j**). Data are expressed as mean ± standard deviation. Two-way analysis of variance was performed to assess the statistical interaction between age and injury (**i**,**j**). Since a significant statistical interaction between age and injury affected the number of LOX (+) cells in the ECM, a multiple-group comparison with Tukey’s honest significant difference test was performed (**j**). Statistical significance is indicated by *** *p* < 0.0001. Individual data points are indicated by circles in the graph; CTX: cardiotoxin. The right tibialis anterior muscle of young mice was designated as Young + CTX, and the left tibialis anterior muscle was designated as Young + Saline. The right tibialis anterior muscle of old mice was designated as Old + CTX, and the left tibialis anterior muscle was designated as Old + Saline.

LOX localization in TA cross-sections was observed using IHC with an anti-LOX antibody (Figure 9a–h); LOX-IR in muscle fibers was observed in nuclei and cytoplasm and was also identified as punctures in the ECM region in all groups (arrowheads and arrows in Figure 9e–h). In previous studies, LOX is produced by fibroblasts, endothelial cells, and muscle fibers and is known to localize to the ECM and muscle fibers [20], and similar results were observed in the present study. After muscle injury, LOX (+) muscle fibers and LOX (+) cells in the ECM were highly expressed in the regenerating muscle of both young and old mice (arrowheads and arrows in Figure 9f,h). The number of LOX (+) muscle fibers increased after muscle injury in both young and old mice (Figure 9i). In addition, cells in which LOX and hematoxylin co-localized in the ECM region were counted as LOX (+) cells (Figure 9j). The results showed significant interactions between age and injury, and multi-group comparisons were made. The number of LOX (+) cells in the ECM of the Old + CTX group was higher than that of the Old + Saline and Young + CTX groups. These results suggest that the number of LOX (+) muscle fibers increases after muscle injury in both young and old mice, but aging may enhance the increase in the number of LOX (+) cells in the ECM region.

## 3. Discussion

In this study, we investigated the effects of aging on collagen remodeling and expression and localization of LOX in skeletal muscles during the recovery period after muscle injury. The salient findings of this study are discussed here. First, *Col1a1* expression as well as collagen I-IR intensity and area were significantly increased during recovery after muscle injury in old mice compared with those in young mice. Second, the expression of *Mmp9* and MMP9 after muscle injury was higher in old mice than in young mice. Third, the expression of *Lox* was significantly increased during recovery after muscle injury in old mice compared with that in young mice. Fourth, the expression of LOX was elevated after muscle injury in both old and young mice. Fifth, the number of LOX (+) muscle fibers and LOX (+) cells in the ECM increased after injury in both the old and young groups, and the number of LOX (+) cells in the ECM after injury was higher in old mice than in young mice. Overall, these results indicate that collagen I may accumulate after muscle injury in old age, accompanied by increased MMP9 expression. In addition, the increase in the expression of LOX gene and the number of LOX (+) cells in the ECM region during muscle recovery in old age compared with that in young age may reflect the formation of collagen cross-links in aging muscle that poses difficulty in muscle recovery. These findings on the expression of LOX gene and LOX localization characterize the effects of aging on intramuscular collagen remodeling and are among the new findings of this study.

During the recovery process after muscle injury in old mice, collagen I accumulates in the perimysium and endomysium of muscles. Collagen expression during muscle recovery is considered a normal healing response [23]. However, in severe injuries or challenging healing conditions, fibrotic collagen accumulates at the injury site, leading to fibrosis [24,25]. In this study, collagen I-IR intensity and area were expanded in the endomysium and perimysium during the recovery process in old mice, whereas young mice showed smoother recovery with less pronounced responses. These findings suggest that healing of CTX-induced muscle injury may be more challenging in old age, potentially resulting in fibrosis.

MMP9 expression may be accentuated in aged muscles during recovery after muscle injury. In this study, gene induction of *Mmp2* and *Mmp9* and protein expression of MMP9 were significantly increased during muscle recovery in old age. MMP2 and MMP9 are enzymes that act on various ECM components, including collagens I and IV, which are crucial for ECM remodeling [15,16,26]. Therefore, the increase in MMPs during muscle recovery can be interpreted as part of the response to intramuscular ECM repair. In fact, upregulation of MMP2 and MMP9 expression has been reported in the muscles of young mice after muscle injury [27]. Furthermore, in the fragile muscle of mdx mice, constant MMP2 upregulation is thought to reflect necrosis and regeneration associated with muscle injury [27]. Moreover, studies have indicated MMP overexpression in aging skin, leading to collagen fibril damage and disorganization, which contributes to skin wrinkling and reduced elasticity, thereby adversely affecting tissue structure and function [26,28,29,30,31]. These findings suggest that high MMP expression during muscle recovery in old age may indicate refractory ECM remodeling.

In this study, we show for the first time that aging affects gene induction of LOX during the recovery process after muscle injury. The expression of LOX and LOXL1 genes in the skin and aorta of rats has been reported to decrease with aging [32,33,34,35]. Although the tissues and model animals used in these previous studies differed from those used in the present study, a trend toward decreased expression of LOX and LOXL1 genes with aging was also observed in the present study. Moreover, in the present study, the expression of the LOX gene was increased in old mice compared with that in young mice during the process of recovery after muscle injury. This finding indicates that the ability to upregulate the expression of the LOX gene after tissue injury may be retained in old age and is one of the novel findings of this study. A previous study has reported that LOX participates in collagen cross-link formation [36]. Indeed, in culture experiments using periodontal ligament cells, it was suggested that gene induction of LOX is induced by mechanical stimulation and contributes to ECM stabilization [37]. Furthermore, gene induction of LOX is known to be elevated in fibrosis of various organs [18]. These findings suggest that gene induction may occur not only in ECM stabilization but also in pathological conditions where excessive collagen accumulation occurs in association with collagen cross-link formation. In a skeletal muscle study, *Lox* expression is upregulated in dystrophic muscle with fibrosis in mdx mice, suggesting that collagen cross-link formation is promoted within dystrophic muscle [20]. Furthermore, *Lox* expression has been reported to correlate with *Col1a1* and *Col3a1* expression [38]. In the present study, both *Lox* and *Col1a1* expression were elevated in aging muscle during the recovery process from injury compared to young mice, which may reflect collagen cross-link formation in injured old muscle.

In this study, we also show that aging may enhance the increase in the number of LOX (+) cells in the ECM during the recovery process after muscle injury. We found that LOX expression was increased after muscle injury in both young and old mice. This may reflect the importance of LOX expression in the muscle recovery process, as LOX expression was also found to increase in the fragile muscles of mdx mice, which are always prone to muscle injury [20]. Furthermore, in the present study, we observed that LOX (+) muscle fibers are less abundant in mature muscle fibers in the TA but are highly expressed in regenerating TA muscle. This result shows the same trend as in previous studies [2,39,40] and reaffirms that the experiments in this study are effective. We found for the first time that the number of LOX (+) cells in the ECM region increases with age after muscle injury. Previous studies have reported increased LOX-IR in the skin of patients with systemic scleroderma, a disease characterized by fibrosis [41]. In the edematous phase of systemic scleroderma, LOX staining was increased in the intra- and extracellular dermis, indicating that LOX is highly expressed in skin lesions where fibrosis occurs. Although the tissues and pathological conditions targeted in the previous studies differed from those in the present study, these findings suggest that the expression of LOX may be increased intra- and extracellularly in the skeletal muscle as well as in the skin under pathological conditions where fibrosis occurs. Furthermore, in this study, the increased number of LOX (+) cells in the ECM region during muscle recovery in old age compared with that in young age may be a characteristic effect of aging on muscle recovery. LOX is known to be produced by fibroblasts, endothelial cells, and muscle fibers [39,42,43]. A previous study has suggested that LOX produced by muscle fibers contributes to muscle differentiation, while LOX in the ECM region contributes to fibrosis [40]. In fact, in the present study, we observed an overaccumulation of collagen I in injured muscle in old mice. These results suggest that an increase in LOX (+) cells in the ECM region may induce fibrosis in the injured muscle of old mice.

Although relevant findings were obtained, this study has several limitations. First, only male mice were used in this study, and sex-related differences in muscle recovery and collagen synthesis were not investigated. In a previous study, the recovery of muscle fiber size after drug-induced muscle injury is faster in females than in males [44]. Furthermore, the amount of collagen in total protein was reported to be higher in males than in females at the same age throughout the mouse body [45]. Given these previous findings, the influence of sex on collagen remodeling during recovery from muscle damage in old age needs to be investigated in the future. Second, the timing of the study was limited to one week after recovery, and the process of healing of the injured muscle was not tracked over time. In previous studies, CTX-injured muscles in young mice showed reduction in fibrosis after a one-month recovery period [46]. It is not known whether injured muscles in old mice can recover after a prolonged recovery period. However, in the present study, fibrosis was observed in injured aged muscles at the one-week recovery stage, compared with the smooth recovery in young mice. Furthermore, inadequate healing is known to develop into irreversible fibrosis [47]. Some intervention may be necessary to promote the recovery of aging muscles. For example, some antifibrotic drugs and stretching are known to inhibit fibrosis in muscle [48,49]. Further research is needed on drug and exercise therapies to promote the recovery of injured muscle in old mice.

## 4. Materials and Methods

### 4.1. Animals

The experiments were conducted using male C57BL/6J mice (Jackson Laboratory, Kanagawa, Japan) aged 21 weeks (young; n = 6) and 92 weeks (old; n = 6). All animals were housed in conventional transparent plastic cages with unrestricted access to food and water. The environmental temperature was controlled at 22 ± 2 °C, with a 12-h light/dark cycle. Approval for the study protocol was granted by the Animal Care and Use Committee of Hokuriku University (approval number: 23-14; approval date: 10 April 2023). All procedures adhered to the Institutional Guidelines for the Use of Laboratory Animals and the ARRIVE guidelines.

### 4.2. Grip Strength Test

One week prior to the start of the experiment, a grip strength test was performed to evaluate muscle function. Grip strength of all four limbs was measured using a grip strength meter (GPM-101B; Melquest, Toyama, Japan). Each mouse was placed on a metal grid attached to the grip strength meter, and each mouse’s tail was gently pulled manually until the limb was released. Peak grip strength (N) was measured three times for each mouse. Time intervals were set at 1 min. The mean of the three trials was used for statistical analysis.

### 4.3. Muscle Injury and Sampling

Muscle injury was induced in the right TA muscle of mice by intramuscular injection of 10 μM CTX (100 μL) as described previously [24,27]. The left TA was injected with the same volume of saline as a sham treatment. A recovery period of one week after intramuscular injection was provided. The right TA of young mice was designated as Young + CTX, and the left TA was designated as Young + Saline. The right TA of old mice was designated as Old + CTX, and the left TA was designated as Old + Saline. One week following the intramuscular injections, the mice were weighed and euthanized using cervical dislocation. The TA muscles were dissected and weighed. To ensure optimal preservation for subsequent analyses, a portion of each TA muscle was immediately immersed in RNAlater (Thermo Fisher Scientific, Waltham, MA, USA). The remaining tissue was rapidly frozen in pre-cooled isopentane and stored at −80 °C for further biochemical and histological investigations.

### 4.4. Histochemical Analysis

Transverse sections (10 μm thick) of the TA muscle were obtained from the middle portion using a cryostat (CM1950; Leica, Wetzlar, Germany) at −25 °C. The tissue sections were mounted on glass slides and processed for HE staining. For the staining procedure, the sections were first incubated with hematoxylin for 10 min to stain the nuclei, followed by eosin for 1 min. After staining, the sections were dehydrated using a graded ethanol series, cleared in xylene, and then mounted in Permount Mounting Medium (Falma Inc., Tokyo, Japan). The stained sections were examined under a microscope (BZ-X810; Keyence, Osaka, Japan).

### 4.5. Quantitative PCR

Total RNA was extracted from the tissue samples using TRIzol reagent (Thermo Fisher Scientific, Waltham, MA, USA). The quality of the extracted RNA was assessed, and the RNA concentration was subsequently normalized to 0.5 μg for further analysis. First-strand complementary DNA (cDNA) synthesis was performed using random primers in conjunction with ReverTra Ace (Toyobo, Osaka, Japan). Quantitative PCR was conducted using TB Green Premix Ex Taq II (Takara Bio, Shiga, Japan) and the StepOnePlus Real-Time PCR System (Thermo Fisher Scientific, Waltham, MA, USA). The following thermocycling conditions were applied: an initial cycle at 95 °C for 30 s, followed by 40 cycles of 95 °C for 5 s and 60 °C for 30 s. A standard calibration curve was generated using the cDNA template, allowing for the quantification of each target gene. The expression levels of the target genes were normalized to those of the housekeeping gene, 18S ribosomal RNA (*Rn18s*). Upregulation or downregulation of the target genes was determined by comparing their expression levels relative to the Young + Saline group. A detailed list of the primers used in the present study is provided in Table 1.

### 4.6. IHC

Transverse sections of TA muscle, 10 µm in thickness, were obtained from the middle portion using a cryostat (CM1950; Leica, Wetzlar, Germany) set to −25 °C and mounted onto glass slides. The sections were fixed in 4% paraformaldehyde, followed by rinsing with phosphate-buffered saline (PBS; pH 7.4). To block endogenous peroxidase activity, the sections were treated with 3% hydrogen peroxide (H_2_O_2_), rinsed again with PBS, and incubated with PBS containing 1% normal goat serum and 0.3% Triton X-100 at 4 °C for 1 h. The sections were incubated overnight at 4 °C with a rabbit polyclonal anti-collagen I or LOX antibody (ab21286 and ab174316; Abcam, Cambridge, MA, USA) diluted 1:500 in PBS with 0.3% Triton X-100. Following primary antibody incubation, the sections were treated with biotinylated anti-rabbit immunoglobulin G (Vectastain ABC kit; Vector Laboratories, Burlingame, CA, USA) at a dilution of 1:1000 in PBS for 1 h at room temperature (25 °C), followed by incubation with avidin-biotin complex (Vectastain ABC kit) for 1 h at 4 °C. After washing with PBS, the sections were rinsed with Tris-HCl buffer (pH 7.4) and incubated in a diaminobenzidine solution (0.035%) in Tris-HCl buffer containing 0.001% H_2_O_2_ for 15 min at 25 °C. Upon completion of the diaminobenzidine reaction, the sections were counterstained with hematoxylin, dehydrated using a graded ethanol series, cleared in xylene, and mounted using Permount™ Mounting Medium (Falma Inc., Tokyo, Japan).

### 4.7. Morphological Analysis

Areas injured by CTX or saline injection were measured with ImageJ Fiji using HE-stained images [50]. The region with either central nucleus fibers or necrotic fibers was defined as the area where the muscle injury occurred because one week after the muscle injury is the phase of regeneration [51]. The percentage of the injured area in the entire transverse section was calculated. In addition, HE-stained images were analyzed using the Analysis Application Hybrid cell count and Macrocell count according to the BZ-X810 microscope instruction manual (Keyence, Osaka, Japan) to determine the FCSA of intact muscle fibers in Young + Saline and Old + Saline groups and the FCSA of fibers with central nuclei in Young + CTX and Old + CTX groups. FCSA analysis followed methods from previous studies [52,53], measuring over 150 muscle fibers per sample. Regarding image analysis in IHC, a semi-quantitative analysis was conducted to assess the intensity of collagen I-IR [54]. Each image had an area of 393,880 μm^2^, and two images per TA muscle were analyzed using ImageJ Fiji (version: 2.14.0/1.54f) [50]. The photographs included the endomysium and perimysium. The collagen I-IR area was also measured with same photographs using the Analysis Application Hybrid cell count according to the BZ-X810 microscope instruction manual (Keyence, Osaka, Japan). The measured collagen I-IR area was calculated as a percentage per area. Furthermore, the number of LOX (+) cells in ECM and LOX (+) muscle fibers was counted using two photographs with an area of 393,880 μm^2^ each. Cells co-expressing LOX and hematoxylin in ECM were designated as LOX (+) cells in ECM. Muscle fibers in which LOX-IR was observed in the cytoplasm or nucleus, or both, were designated as LOX (+) muscle fibers. In addition, images were analyzed in a single-blinded manner.

### 4.8. WB

The expression levels of LOX, MMP2, and MMP9 were assessed using WB. Frozen muscle tissues were homogenized in an ice-cold homogenization buffer containing 50 mM Tris-HCl, 150 mM NaCl, 0.1% SDS, 1% NP-40, and 0.5% sodium deoxycholate (pH 8.0), supplemented with a protease inhibitor cocktail (FUJIFILM Wako Pure Chemical Corporation, Osaka, Japan). The homogenates were centrifuged at 15,000 rpm for 5 min at 4 °C, and the resulting supernatant was carefully collected for further analysis. Protein concentrations were determined using the DC protein assay kit (Bio-Rad Laboratories, Hercules, CA, USA). The supernatant was then solubilized in SDS sample buffer (10% glycerol, 2% SDS, 0.005% bromophenol blue, 100 mM dithiothreitol, and 50 mM Tris-HCl, pH 6.8), followed by heating at 98 °C for 3 min to ensure denaturation of the proteins. Subsequently, equal amounts of protein were resolved by electrophoresis on 8% (for MMP2 and 9), 10% (for GAPDH), or 12% (for LOX) sodium dodecyl sulfate-polyacrylamide gels (SDS-PAGE) and transferred to polyvinylidene difluoride membranes. To block nonspecific binding, the membranes were incubated in 5% skim milk dissolved in Tris-buffered saline with 0.1% Tween 20. Membranes were then probed with primary antibodies, including rabbit monoclonal anti-LOX (ab 174316; Abcam, Cambridge, MA, USA), rabbit monoclonal anti-MMP2 (ab92536; Abcam, Cambridge, MA, USA), or rabbit polyclonal anti-MMP9 (10375-2-AP; Proteintech Group, Inc., Rosemont, IL, USA), and incubated overnight at 4 °C. Following the incubation with primary antibodies, the membranes were incubated with horseradish peroxidase-conjugated secondary antibodies (Cell Signaling Technology, Beverly, MA, USA) for one additional hour at room temperature. GAPDH (ab181602; Abcam, Cambridge, MA, USA) was used as an internal loading control to ensure equal protein loading. Immunoreactive signals were visualized using a chemiluminescence detection system (Immunostar Zeta; FUJIFILM Wako Pure Chemical Corporation, Osaka, Japan), and the intensity of the signals was quantified with an image reader (FUSION Solo7; M&S Instruments Inc., Osaka, Japan). The digitized signal intensities were analyzed using ImageJ Fiji software (version: 2.14.0/1.54f) [50].

### 4.9. Statistical Analysis

Statistical analyses were conducted using EZR (Saitama Medical Center, Jichi Medical University, Saitama, Japan) [55], a user interface for R (R Foundation for Statistical Computing, Vienna, Austria). EZR is a customized version of the R Commander that includes tools for statistical methods frequently used in the field of biostatistics. To assess the effects of aging and muscle injury, a two-way analysis of variance was applied. If a significant interaction between the factors was identified, Tukey’s honest significant difference test was used for post hoc pairwise comparisons across all groups. Statistical significance was set at a *p* < 0.05. Results are presented as means ± standard deviations. In addition, the probability of significance by the Shapiro–Wilk test was compared to the adjusted significance level using the Holm method in this study. Normality was confirmed in the experimental data (4-group comparison). Since normality was not confirmed in the experimental data (2-groups comparison) only in the data of the injured area by CTX, the Mann–Whitney U test was used to compare the groups. For all other data, the Student’s *t*-test was used for 2-group comparisons. Furthermore, the results of the Shapiro–Wilk test conducted to confirm normality are expressed as adjusted *p*-values using the Holm method (Tables S1 and S2).

## 5. Conclusions

In the present study, *Col1a1* expression as well as collagen I-IR intensity and area during recovery after muscle injury were significantly higher in old mice compared to young ones. Similarly, *Lox* expression and the number of LOX (+) cells in the ECM during recovery were also significantly increased in old mice. Furthermore, expression levels of *Mmp2*, *Mmp9,* and MMP9 after muscle injury were higher in old mice than in young mice. These results suggest that collagen I accumulation and collagen cross-link formation may occur after muscle injury in old age, along with increased MMP9 expression.

## Figures and Tables

**Table 1:** Primer sequences utilized in the present study.

Genes	Direction	Nucleotide Positions and Sequence (5′-3′)	Sequence ID
*Col1a1*	Forward	2916 GATCTCCTGGTGCTGATG 2933	NM_007742.4
	Reverse	3028 GAAGCCTCTTTCTCCTCTCTGA 3007	NM_007742.4
*Col3a1*	Forward	3587 CAGGTCCTAGAGGAAACAGA 3606	BC052398.1
	Reverse	3728 TCACCTCCAACTCCAACAATG 3708	BC052398.1
*Mmp2*	Forward	2120 AAGAAAATGGACCCCGGTTT 2139	NM_008610.3
	Reverse	2251 CTTCAGGTAATAAGCACCCTTG 2230	NM_008610.3
*Mmp9*	Forward	969 CAGCCAACTATGACCAGGAT 988	NM_013599.5
	Reverse	1217 CTGCCACCAGGAACAGG 1201	NM_013599.5
*Lox*	Forward	890 GTGCCCGACCCCTACTACAT 909	M65142.1
	Reverse	1007 TGACATCCGCCCTATATGCT 988	M65142.1
*Loxl1*	Forward	2280 GGCCTCAGGGAGTGAACATG 2299	NM_010729.3
	Reverse	2339 AAGACAGGGTCTGGCATCCA 2320	NM_010729.3
*Loxl2*	Forward	3257 CCTCCCTCCCGCTTTCA 3273	NM_033325.2
	Reverse	3313 CAAGTGTGCAGTCCTGGGTTT 3293	NM_033325.2
*Loxl3*	Forward	2603 CCCCAGCAACAGACAGAACA 2622	NM_013586.5
	Reverse	2661 GAGCTGCTGCCATCCTGTGT 2642	NM_013586.5
*Loxl4*	Forward	3501 GCAGCTTCCACTGCACTACACT 3522	NM_001164311.1
	Reverse	3561 TGTTCCGAGCGTCATCCA 3544	NM_001164311.1
*Rn18s*	Forward	1617 GCAATTATTCCCCATGAACG 1636	NR_003278.3
	Reverse	1739 GGCCTCACTAAACCATCCAA 1720	NR_003278.3

## Data Availability

The data presented in this study are available on figshare as follows. Muscle injury: https://doi.org/10.6084/m9.figshare.27411360.v3, uploaded on 1 November 2024 (Posted date: 27 November 2024). Hematoxylin and eosin_Young (21 w): https://doi.org/10.6084/m9.figshare.27266223.v5, uploaded on 21 October 2024 (Posted date: 6 December 2024). Hematoxylin and eosin_Old (92 w): https://doi.org/10.6084/m9.figshare.27266238.v3, uploaded on 21 October 2024 (Posted date: 6 December 2024). Collagen I localization_Young (21 w): https://doi.org/10.6084/m9.figshare.27252993.v6, uploaded on 18 October 2024 (Posted date: 6 December 2024). Collagen I localization_Old (92 w): https://doi.org/10.6084/m9.figshare.27253005.v4, uploaded on 18 October 2024 (Posted date: 6 December 2024). LOX localization_Young (21 w): https://doi.org/10.6084/m9.figshare.27966486.v1, uploaded on 5 December 2024 (Posted date: 5 December 2024). LOX localization_Old (92 w): https://doi.org/10.6084/m9.figshare.27966837.v1, 5 December 2024 (Posted date: 5 December 2024). LOX, MMP, and GAPDH expression: https://doi.org/10.6084/m9.figshare.27274878.v5, uploaded on 22 October 2024 (Posted date: 6 December 2024).

## Supplementary Materials

The following supporting information can be downloaded at: https://www.mdpi.com/article/10.3390/ijms26020801/s1.
