# Supplementary material for: Effects of Aging on Intramuscular Collagen-Related Factors After Injury to Mouse Tibialis Anterior Muscle
Source: Int J Mol Sci. 2025 Jan 18;26(2):801. doi: 10.3390/ijms26020801 (PMC11766099; doi:10.3390/ijms26020801)
Supplement: Supplementary file 1 [file ijms-26-00801-s001.zip › Supplementary File/Table S2.docx]

| Table S2. Confirmation of normality (2-groups comparison) | | |  |
| --- | --- | --- | --- |
| Group | Experimental data | Adjusted *p*-value |  |
| Old | Body weight | 0.911 |  |
| Old | Body weight before experiment | 2.114 |  |
| Old | Grip strength | 0.256 |  |
| Old | Grip strength/body weight | 1.603 |  |
| Old | Injured area (Saline) | 2.168 |  |
| Old | Injured area (CTX) | 0.090 |  |
| Old | LOX expression (CTX) | 1.628 |  |
| Young | Body weight | 1.904 |  |
| Young | Body weight before experiment | 2.111 |  |
| Young | Grip strength | 2.132 |  |
| Young | Grip strength/body weight | 1.426 |  |
| Young | Injured area (Saline) | 0.747 |  |
| Young | Injured area (CTX) | 0.002 |  |
| Young | LOX expression (CTX) | 0.183 |  |
| The *p*-values by the Shapiro-Wilk test were presented as adjusted *p*-values by the Holm method. | | |  |
|  |  |  |  |
