# Supplementary material for: Effects of Aging on Intramuscular Collagen-Related Factors After Injury to Mouse Tibialis Anterior Muscle
Source: Int J Mol Sci. 2025 Jan 18;26(2):801. doi: 10.3390/ijms26020801 (PMC11766099; doi:10.3390/ijms26020801)
Supplement: Supplementary file 1 [file ijms-26-00801-s001.zip › Supplementary File/Table S1.docx]

| Table S1. Confirmation of normality (4-groups comparison) | | |  |
| --- | --- | --- | --- |
| Group | Experimental data | Adjusted *p*-value |  |
| Young + Saline | *Col1a1* expression | 13.967 |  |
| Young + Saline | *Col3a1* expression | 10.408 |  |
| Young + Saline | Collagen I-IR area | 11.459 |  |
| Young + Saline | Collagen I-IR intensity | 1.467 |  |
| Young + Saline | FCSA | 6.857 |  |
| Young + Saline | LOX (+) cells in ECM | 2.895 |  |
| Young + Saline | LOX (+) muscle fiber | 12.514 |  |
| Young + Saline | *Lox* expression | 5.297 |  |
| Young + Saline | *Loxl1* expression | 10.691 |  |
| Young + Saline | *Loxl2* expression | 13.384 |  |
| Young + Saline | *Loxl3* expression | 3.845 |  |
| Young + Saline | *Loxl4* expression | 0.998 |  |
| Young + Saline | *Mmp2* expression | 11.845 |  |
| Young + Saline | MMP2 expression | 10.521 |  |
| Young + Saline | *Mmp9* expression | 8.379 |  |
| Young + Saline | MMP9 expression | 12.056 |  |
| Young + Saline | Muscle weight | 13.736 |  |
| Young + CTX | *Col1a1* expression | 10.220 |  |
| Young + CTX | *Col3a1* expression | 12.532 |  |
| Young + CTX | Collagen I-IR area | 1.999 |  |
| Young + CTX | Collagen I-IR intensity | 1.037 |  |
| Young + CTX | FCSA | 13.907 |  |
| Young + CTX | LOX (+) cells in ECM | 12.136 |  |
| Young + CTX | LOX (+) muscle fiber | 12.055 |  |
| Young + CTX | *Lox* expression | 8.915 |  |
| Young + CTX | *Loxl1* expression | 12.859 |  |
| Young + CTX | *Loxl2* expression | 13.581 |  |
| Young + CTX | *Loxl3* expression | 9.164 |  |
| Young + CTX | *Loxl4* expression | 11.402 |  |
| Young + CTX | MMP2 expression | 13.035 |  |
| Young + CTX | *Mmp2* expression | 9.508 |  |
| Young + CTX | MMP9 expression | 13.023 |  |
| Young + CTX | *Mmp9* expression | 13.770 |  |
| Young + CTX | Muscle weight | 13.668 |  |
| Old + Saline | *Col1a1* expression | 3.981 |  |
| Old + Saline | *Col3a1* expression | 12.480 |  |
| Old + Saline | Collagen I-IR area | 12.367 |  |
| Old + Saline | Collagen I-IR intensity | 11.142 |  |
| Old + Saline | FCSA | 4.682 |  |
| Old + Saline | LOX (+) cells in ECM | 9.874 |  |
| Old + Saline | LOX (+) muscle fiber | 13.920 |  |
| Old + Saline | *Lox* expression | 8.719 |  |
| Old + Saline | *Loxl1* expression | 5.933 |  |
| Old + Saline | *Loxl2* expression | 14.207 |  |
| Old + Saline | *Loxl3* expression | 12.964 |  |
| Old + Saline | *Loxl4* expression | 0.484 |  |
| Old + Saline | MMP2 expression | 11.443 |  |
| Old + Saline | *Mmp2* expression | 5.248 |  |
| Old + Saline | MMP9 expression | 2.899 |  |
| Old + Saline | *Mmp9* expression | 13.070 |  |
| Old + Saline | Muscle weight | 13.243 |  |
| Old + CTX | *Col1a1* expression | 13.398 |  |
| Old + CTX | *Col3a1* expression | 5.609 |  |
| Old + CTX | Collagen I-IR area | 11.195 |  |
| Old + CTX | Collagen I-IR intensity | 14.063 |  |
| Old + CTX | FCSA | 7.561 |  |
| Old + CTX | LOX (+) cells in ECM | 12.883 |  |
| Old + CTX | LOX (+) muscle fiber | 3.343 |  |
| Old + CTX | *Lox* expression | 6.010 |  |
| Old + CTX | *Loxl1* expression | 10.434 |  |
| Old + CTX | *Loxl2* expression | 4.926 |  |
| Old + CTX | *Loxl3* expression | 13.873 |  |
| Old + CTX | *Loxl4* expression | 14.054 |  |
| Old + CTX | MMP2 expression | 1.964 |  |
| Old + CTX | *Mmp2* expression | 12.385 |  |
| Old + CTX | MMP9 expression | 13.183 |  |
| Old + CTX | *Mmp9* expression | 10.499 |  |
| Old + CTX | Muscle weight | 13.876 |  |
| The *p*-values by the Shapiro-Wilk test were presented as adjusted *p*-values by the Holm method. | | |  |
|  |  |  |  |
